# Introduction to Noise Radar and Its Waveforms

**DOI:** 10.3390/s20185187

**Published:** 2020-09-11

**Authors:** Francesco De Palo, Gaspare Galati, Gabriele Pavan, Christoph Wasserzier, Kubilay Savci

**Affiliations:** 1 Department of Electronic Engineering, Tor Vergata University of Rome, now with Rheinmetall Italy, 00131 Rome, Italy; fradepalo89@gmail.com; 2 Department of Electronic Engineering, Tor Vergata University and CNIT-Consortium for Telecommunications, Research Unit of Tor Vergata University of Rome, 00133 Rome, Italy; gaspare.galati@uniroma2.it; 3 Fraunhofer Institute for High Frequency Physics and Radar Techniques FHR, 53343 Wachtberg, Germany; christoph.wasserzier@fhr.fraunhofer.de; 4 Turkish Naval Research Center Command and Koc University, Istanbul, 34450 İstanbul, Turkey; ksavci@ieee.org

**Keywords:** noise radar technology, noise waveforms, peak sidelobe level, ambiguity function, waveform design, peak-to-average-power-ratio

## Abstract

In the system-level design for both conventional radars and noise radars, a fundamental element is the use of waveforms suited to the particular application. In the military arena, low probability of intercept (LPI) and of exploitation (LPE) by the enemy are required, while in the civil context, the spectrum occupancy is a more and more important requirement, because of the growing request by non-radar applications; hence, a plurality of nearby radars may be obliged to transmit in the same band. All these requirements are satisfied by noise radar technology. After an overview of the main noise radar features and design problems, this paper summarizes recent developments in “tailoring” pseudo-random sequences plus a novel tailoring method aiming for an increase of detection performance whilst enabling to produce a (virtually) unlimited number of noise-like waveforms usable in different applications.

## 1. Introduction

Noise radar technology (NRT) is based on the transmission of random waveforms as opposed to the classical, often sophisticated, deterministic radar signals [1,2]. Both NRT and “deterministic radar” technology use the “matched filter”, or an approximation of it, to maximize the signal-to-noise ratio (SNR). In a conventional radar, a single waveform (or a finite set of waveforms), is used for transmission and reception with matched filtering. Conversely, NRT is able to transmit a—virtually unlimited—set of realizations (i.e., “sample functions”) of a random process, with the pertaining matched filter set up in real time to implement the well-known “correlation receiver”. NRT was conceived long ago [3], but its practical interest revamped around the 2000s with the advancements in digital acquisition, processing, and generation of signals plus, in the military sector, with the increasing need for low probability of intercept (LPI) radars. The main advantages of NRT are recalled here.

- Hard, if not impossible, exploitation of the transmitted signals by an enemy party aimed to intercept, classify, identify (and possibly jam) the radar. The low probability of intercept, i.e., the reduced visibility of radar signals by ESM (electronic support measures) and ELINT (electronic intelligence) systems [4] is significantly enhanced by the random nature of NRT signals. The low probability of intercept and low probability of exploitation by a hostile counterpart will be analysed in more detail in a companion paper to be submitted on the counter-interception and counter-exploitation features of NRT.
- Limitation of the mutual interferences to/from many radars located in a crowded environment and operating in a limited frequency band. In fact, using NRT the interfered radar perceives and treats the interference as a kind of noise [5,6]. This feature will be more and more important in the developments of marine radars, due to their use of solid-state transmitters, having much greater “duty cycle” (longer pulses) than the legacy magnetron [7], as well as in automotive applications, [8] because of the more and increasing number of used automotive radars, causing a high spatial density of these particular radar sets.
- Favourable shape of the “ambiguity function”. The ambiguity function [1,2] of a radar waveform defines its “selectivity” both in distance, i.e., in radar range (proportional to the time delay) and in radial velocity (proportional to the frequency shift due to the Doppler effect). The ambiguity function of a conceptual waveform made up by an infinitely long realization of “white noise” (i.e., with duration T and bandwidth B both going to infinity) tends to a Dirac’s “delta-shape” function with an ambiguity level going to zero. However, in all real applications both B and T are limited, and the peak side lobe ratio (PSLR) of the ambiguity function has a mean value close to the “processing gain” B·T (hereafter, BT). Both bandwidth and duration are practically limited. The limitations on B come from many factors, including the constraints on the usable portion of the electromagnetic spectrum [9]. Those on T depend (i) on the dynamic behaviour (“decorrelation time”, $T_{C}$) of the radar cross section of most targets and (ii) on the dwell time, $T_{D}$, and the pertaining type of radar processing. Echoes from moving targets (vehicles) have some typical decorrelation time $T_{C}$ (related to the signal fluctuation) of the rough order of hundreds of milliseconds. Doppler effect may pose a further limitation on the coherent signal integration time T, imposed to be smaller than both $T_{C}$ and $T_{D}$, and typically in the order of tens or hundreds of milliseconds. With an exemplary bandwidth of 50 MHz and an exemplary value of T=20 ms, the processing gain of a continuous-emission (CE) noise radar is of the order of one million. The resulting average PSLR is about 60 dB, which is acceptable in many radar applications, when the “fourth power of distance” term does not affect too much the needed dynamic range.

**Figure 1:**
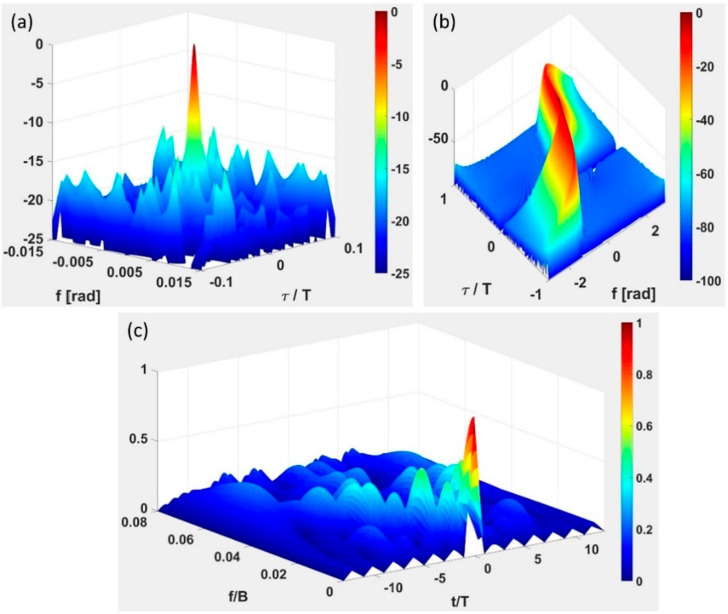
Ambiguity function (values in dB for (**a**,**b**) and in linear scale for (**c**)): (**a**) Noise waveform (BT = 100); (**b**) non-linear frequency modulated (NLFM) “chirp” signal (BT = 100), and (**c**) 13-elements Barker code. The low and impractical BT values are here chosen for the clarity of display.

A comparison between the ambiguity function of a typical application of the NRT (Figure 1a) and of two deterministic radar signals (non-linear frequency modulation (NLFM), Figure 1b, and 13-elements Barker code [2], Figure 1c) are shown in Figure 1.

Concerning practical radar implementations, the disadvantages of NRT are mainly due to the use of a real-time correlation receiver/processor, with a burden, for computation and analog-to-digital conversion, significantly greater than a classical frequency modulated, continuous wave (FM-CW) radar.

The sampling rate of digital correlation in NRT needs to cover the full frequency band, e.g., 50 MHz when a resolution of 3 m is required. On the other side, FM-CW signals are treated by the de-ramping (or homodyne) receiver principle [10], where the correlation is performed by “de-chirping”, followed by a bank of video filters. This operation is based on beating the received signal with a replica of the transmitted one (a frequency ramp), extracting the baseband spectrum via analog filtering and finally obtaining the correlation; hence, the “Range” of targets, via fast Fourier transform (FFT). This procedure permits the usage of analog-to-digital converters (ADCs) with low sampling rates and high dynamic range, as not the full RF bandwidth but a much narrower base-band converted signal [11] has to be sampled, with a relatively low maximum frequency related to the maximum target distance.

In most FM-CW radars the duration T of the ramp is related to the maximum delay $T_{max}=2R_{max}/c$ (compatible with the maximum range $R_{max}$) by $T_{max}\leq T/\beta$ with β>1. The related energy loss at $R_{max}$ is equal to $L=10\cdot log_{10}\left(1-1/\beta \right)$. To limit L below one dB, β must be not less than five (for β=5, L≅0.97 dB; for β=6, L≅0.8 dB). However, once fixed both B (by the international regulations on the electromagnetic spectrum) and $T_{max}$ ($R_{max}$) by the application, an increase of β; hence, of *T* for the prescribed $T_{max}$, implies a reduction of the slope B/T of the frequency-vs-time law. The resulting increase of the unwanted Range–Doppler coupling makes the FM-CW radar less flexible in terms of choice of *T* than a continuous emission (CE) noise radar.

For a range resolution of 3 m, i.e., a height of the ramp B=50 MHz and a duration T=1 ms (compatible with a maximum range $R_{max}=30$ km, i.e., β=5), the beat frequencies, in the FM-CW case, are between 0 and 10 MHz, i.e., the sampling rate is five times less than the NRT, with and a much cheaper processing than a full correlation. This example clarifies that noise radar technology can be preferred (for example, over the simple FM-CW solution) when there are precise requirements concerning either a high resolution in Range and in Doppler (calling for a “thumbtack-like” ambiguity function of the pseudorandom signals), or the radiation of many “nearly-orthogonal” signals with minimal “cross-correlation” and LPI/LPE features.

Having defined the main signal parameters (duration, bandwidth, …), another remaining flexibility in the system design of a noise radar is the possibility to select the peak-to-average power ratio (PAPR) of each radiated waveform g(t), here defined by its samples g[k] with k=1, 2,…,N, being $f_{s}$ the sampling frequency and $N=f_{s}\cdot T$:

(1) $$PAPR=\frac{\underset{k}{\max}{\left|g\left[k\right]\right|}^{2}}{\frac{1}{N}\sum_{k=1}^{N}{\left|g\left[k\right]\right|}^{2}}$$

The FM-CW signals and the other deterministic waveforms (e.g., chirp, Barker, Polyphase codes, etc. [1,2]) generally have PAPR=1, i.e., exhibit phase/frequency only modulation. Because of their unitary modulus, they can be named “unimodular”. Of course, an arbitrary, or noise, waveform (unless hard-limited, i.e., amplitude saturated) has a PAPR greater than the unity, which depends on its statistics. Amplitude saturation upstream the matched filter reduces the signal energy; hence, causing a detection loss: Table 1 shows the signal-to-noise ratio (SNR) losses as related to the PAPR, i.e.: $Loss\left[\mathrm{dB}\right]=-10\cdot log_{10}\left(PAPR\right).$ Amplitude saturation losses are not present when using a unimodular noise waveform, i.e., when saturation/hard limiting is applied in the waveform generation (i.e., choosing a “phase-only modulation”). However, a unimodular waveform has a reduced number of degrees of freedom, i.e., a number *BT* of phase values in place of 2*BT “*real” degrees of freedom, i.e., amplitude and phase pairs, or equivalently, I and Q (in-phase and quadrature) pairs.

**Figure 2:**
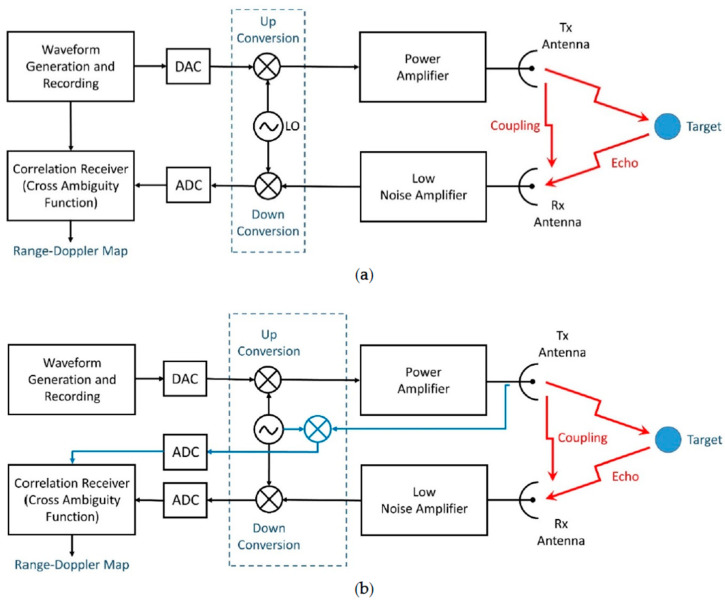
Basic Block Diagram. (**a**) The reference is the digital record of the transmitted code; (**b**) the reference is the record of the transmitted signal at the antenna port; ADC = Analog-to-Digital-Converter; DAC = Digital-to-Analog-Converter.

An effective noise radar operation requires a large enough BT granting: (i) the radiated signals to appear “noise-like”, i.e., random to any hostile counterpart, (ii) high range resolution, and (iii) detectability of small targets in the presence of the Range (and possibly, Doppler) sidelobes of the response of stronger targets at different ranges. These “interfering” targets may have not only a large radar cross section, but also shorter ranges with respect to the wanted targets, causing very strong disturbing returns. Hence, spectrum and time resources have to be fully exploited, leading to a continuous emission (CE) [12] preferred architecture, as shown in Figure 2a,b. Note that in Figure 2 the unavoidable leakage due to antenna coupling has been shown.

The architecture shown in Figure 2b circumvents the problem of the non-ideal transmitting channel but implies the cost of a wideband reference channel and of an additional fast analog to digital convertor (ADC). A final consideration is that continuous emission is needed to best exploit the LPE feature: the absence of the edges of pulses (as well as the random modulation) makes it difficult to extract the signal features. This relevant topic, beyond the scope of this introductory paper, will be treated in an ensuing work.

As anticipated, the “leakage” from the transmitting to the receiving path due to the antenna coupling (Figure 2) is a critical problem with CE operation. This effect is someway similar to a strong target very close to the radar and depends on the antenna system and the RF connections. Coupling affects the radar operation at close or medium distance due to the sidelobes of the autocorrelation function of the radiated waveform. In addition to a careful antenna design, an effective way to counteract this damaging phenomenon is to transmit waveforms with low Range sidelobes.

## 2. Noise Waveforms Properties

### 2.1. Deterministic Waveforms and Noise Waveforms

Concerning the properties of the NRT waveforms, it is useful to clarify the significance of “randomness” in this context. Of course, if randomness is used in its meaning of “unpredictability”, only “pseudorandom” signals may be generated and transmitted. A pseudorandom sequence [13] is defined (in cryptology, for example), as one which passes some statistical tests suited to the particular application. In the military field, such a signal is unknown to the enemy and unpredictable by it.

In principle, noise from physical phenomena (e.g., “thermal noise” in amplifiers or Zener diodes) can be used as a random source. However, ordinary analogue noise generators suffer from important limitations in accuracy/resolution and dynamic range, often making their use impractical and generally not complying with most applications. Hence, as it happens in most cryptographic applications, the preferred implementation of the NRT waveforms is the “digital” one, i.e., using a pseudo-random-number-generator (PRNG) [13]. As the desirable LPI/LPE capabilities of NRT rely on the unpredictability of the signals, in principle this feature would be compromised if pseudo-random sequences should be used instead of “pure noise”. However, nowadays it is easy to generate pseudo-random signals of such complexity, and with a repetition period [13,14], so long as to make signal analysis and reproduction practically unfeasible.

However, a “pure digital noise”, e.g., a Gaussian noise sequence with some suitable spectral density in the radar band, is not always a good choice for NRT systems because of two main problems. First, a “PAPR problem”: most radar transmitters operate in saturation, i.e., emit with constant envelope; the penalty in the radar power budget for transmitting noise waveforms with a relatively large PAPR is shown Table 1 and may be too large for many applications. Secondly, in noise radar the correlation processing of echoes of duration T and bandwidth B generates the well-known “random fluctuations” of the sidelobes as described in the following. In any ordinary radar using a “deterministic” waveform g(t) the peak sidelobe ratio (PSLR) is defined as the ratio between the highest sidelobe of the autocorrelation function (ACF) of g(t) and the maximum value of the ACF. In noise radar, due to the “stochastic” nature of the sidelobes (Section 2.2), we have to define a (statistical) Peak Sidelobe Level PSL, which is not exceeded with a given probability. Such a PSL depend on the particular type of noise waveform and, for the reasons explained in the following, can be estimated as

(2) $$PSL_{dB}=-10\cdot log_{10}\left(BT\right)+K$$

with K being a constant value, depending on the chosen probability and generally in the range 10–12 dB. The statistics of PSL are analysed in Section 2.2.

Nowadays, interest is growing in “tailored” pseudorandom waveforms able to suppress the range sidelobes of the autocorrelation function in a particular delay interval. Powerful algorithms, in particular those said CAN (cyclic algorithm new), are analysed in [15]. Their main limitations are mostly related to the bandwidth constraints often associated to each operating environment. To mitigate these limitations a novel algorithm, named band limited algorithm for sidelobes attenuation (BLASA), has been presented in [16]. A much simpler technique to suppress the sidelobes in a range interval of interest will be described in Section 3 with some results. Its main advantage over CAN and BLASA is their low computational burden, especially for high BT values.

### 2.2. Statistical Properties of Autocorrelation Function, Sidelobes, and Power Spectrum

Let g(t)=X(t)+jY(t) be the complex envelope of a realization of an ergodic, stationary, and band-limited random process. The autocorrelation function (ACF) of g(t) is defined as

(3) $$R\left(\tau \right)=\overset{+\infty }{\underset{-\infty }{\int }}g^{*}\left(t\right)g\left(\tau +t\right)dt$$

The power spectral density (PSD) of the complex envelope, i.e., the Fourier transform of R(τ), is supposed as symmetrical around f=0, with bilateral band B, i.e., from $-\frac{B}{2}$ to $+\frac{B}{2}$. Hence, the real part X(t) and the imaginary part Y(t) of g(t) give an equal (50%) contribution to R(τ), which is a real function. After a maximum delay T/2 we may suppose that R(τ) practically vanishes, being T the “coherent processing interval”, whose value is related to the “time on target” (depending on the antenna rotation rate, and/or on the target velocity) and to the echo decorrelation. In practice T is much greater than the delay of the farthest target $T_{max}$, which defines the maximum range of interest $R_{max}=\frac{cT_{max}}{2}$.

We consider a sampled version of the ACF, i.e., $R\left(kT_{s}\right)≝R\left[k\right]$ obtained by sampling g(t) at frequency $F_{s}=k_{F}\frac{1}{T_{s}}=k_{F}B$ where $k_{F}$ is the oversampling factor. In practical applications it is set $k_{F}>1$, but for the sake of simplicity, we consider here the ideal case $k_{F}=1$. Hence, each waveform at hand is described by a number $N_{F}≝\mathrm{int}\left(B\cdot T\right)\gg 1$ of (theoretically) uncorrelated samples. These $N_{F}$ samples barely contain the information embedded in the random process g(t), i.e., its PSD S(f) and the related Fourier transform R(τ) in the interval $-\frac{T}{2}<\tau <+\frac{T}{2}$.

The sampled version of R(τ) is

(4) $$R\left[k\right]=\overset{+N_{F}/2}{\underset{i=-N_{F}/2}{\sum }}g_{i}^{*}\cdot g_{i+k}\;-\frac{N_{F}}{2}\leq k\leq +\frac{N_{F}}{2}$$

with $\underset{k}{\max}\left\{R\left[k\right]\right\}=R\left[0\right].$ Let us consider the ratio:

(5) $$\rho_{k}≝\frac{R\left[k\right]}{R\left[0\right]}=\frac{\sum_{i=-N_{F}/2}^{N_{F}/2}g_{i}^{*}\cdot g_{i+k}}{\sum_{i=-N_{F}/2}^{N_{F}/2}{\left|g_{i}\right|}^{2}}$$

which of course represents the sample correlation coefficient of the process. For a real process, $\rho_{k}$ is real with values in the range $-1\leq \rho_{k}\leq +1$; the statistical distribution of $\rho_{k}$ has been studied by Fisher [17], who showed that when $N_{F}\gg 1,$ $\rho_{k}$ is approximately Gaussian-distributed with expected value $\eta_{\rho_{k}}≅\rho_{True}$ (i.e., the true correlation coefficient of the generating random process; we do not mention its dependence on k for the sake of simplicity) and variance $\sigma_{\rho_{k}}^{2}≅\frac{1}{N_{F}-3}$. Note that $\rho_{True}$ is the “true value” of the correlation coefficient, while $\rho_{k}$ is the “sample” correlation coefficient.

Defining the absolute value $r=\left|\rho_{k}\right|$, when considering the case of interest in which $\rho_{True}\ll 1$, i.e., the sidelobes region of the autocorrelation function, simple computations show that, for a real signal, the random variable r has an expected value of $\eta_{r}=\sqrt{\frac{2}{\pi }\cdot \frac{1}{N_{F}}}$.

For a complex process g(t), when $\left|\rho_{True}\right|≅1$, it results $Im\left[\rho_{k}\right]≅0$ having supposed the PSD symmetrical around f=0 and $N_{F}\gg 1$. Decreasing $\left|\rho_{True}\right|$, $Im\left[\rho_{k}\right]$ increases and when $\left|\rho_{True}\right|≅0$ (sidelobes region of the ACF) the real and the imaginary part of $\rho_{k}$ give a nearly equal contribution to $\left|\rho_{k}\right|$, being both, approximately, Gaussian-distributed with zero mean value $\left(\rho_{True}≅0\right)$ and same variance $\sigma_{Re\left[\rho_{k}\right]}^{2}=\sigma_{Im\left[\rho_{k}\right]}^{2}≅\frac{1}{2N_{F}}$. Hence, for $\left|\rho_{True}\right|≅0$, the sidelobes have a random behaviour and the output of the correlation filter (we assume that matched filtering is used) is a complex signal similar to receiver noise, i.e., the aforementioned normalized sidelobe level $r=\left|\rho_{k}\right|$ becomes a Rayleigh-distributed random variable with the well-known “probability density function” (PDF) f(r) and the “cumulative distribution function” (CDF) F(r):

(6) $$f\left(r\right)=\;\frac{r}{a_{Ray}^{2}}exp\left(-\frac{r^{2}}{2a_{Ray}^{2}}\right)U\left(r\right)$$

(7) $$F\left(r\right)=\;\left[1-exp\left(-\frac{r^{2}}{2a_{Ray}^{2}}\right)\right]U\left(r\right)$$

where U(·) is the unitary step and $a_{Ray}^{2}≝\frac{1}{2N_{F}}$. In the following, we substitute $N_{F}$ with the more significant product BT; hence, the mean value and the standard deviation of r result:

(8) $$\eta_{r}=\sqrt{\frac{\pi }{4BT}}\;\sigma_{r}=\sqrt{\frac{1}{2BT}\left(2-\frac{\pi }{2}\right)}=\eta_{r}\sqrt{\frac{4}{\pi }-1}$$

Now, we may rewrite the CDF of r, or its complement Q(r)=1−F(r), as a function of the parameter K (in dB) introduced in Equation (2) and rewritten here:

(9) $$r_{K}\left(dB\right)=-10\cdot log_{10}\left(BT\right)+K$$

Equation (9) in linear scale becomes

(10) $$r_{K}=10^{\frac{r_{K\left(dB\right)}}{20}}=\frac{1}{\sqrt{BT}}\cdot 10^{0.05\cdot K}$$

and the resulting probability $P\left\{r>r_{K}\right\}=Q\left(r_{K}\right)$ is

(11) $$Q\left(r_{K}\right)=e^{-\frac{r_{k}^{2}}{2a_{Ray}^{2}}}=e^{-r_{k}^{2}\cdot BT}=e^{-10^{0.1\cdot K}}$$

**Figure 3:**
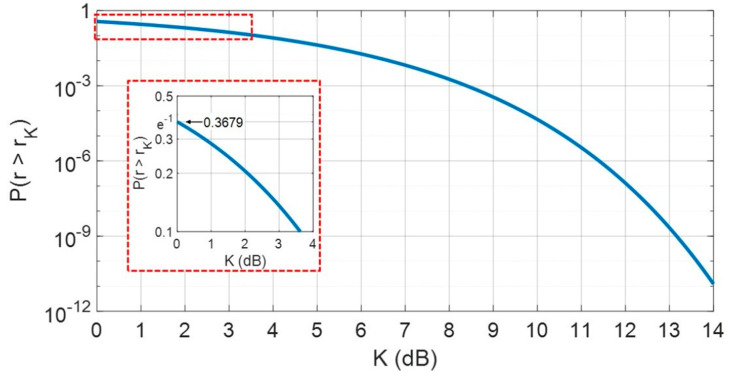
Probability of the event $\left\{r>r_{K}\right\}$, $r_{K}$ given by Equation (11), versus K in dB.

For K=0 dB, it results $P\left\{r\left(dB\right)>-10\cdot log_{10}\left(\mathrm{BT}\right)\right\}=e^{-1}≅0.3679$. Varying K from 0 dB up to 14 dB, Equation (11) is drawn as in Figure 3.

From Equation (11), the Rayleigh CDF of r results:

(12) $$F\left(r_{K}\right)=1-e^{-10^{0.1\cdot K}}$$

Hereafter we will assume BT≫1 and in the sidelobes region (i.e., for $\left|\rho_{True}\right|≅0$) the PSL, i.e., $r_{p}≝\max\left\{r_{K}\right\}$, can be defined as the one having a prescribed probability (hereafter named 1−δ) of not being exceeded in the whole sidelobe region of the ACF (with about $N_{F}≅BT$ sidelobes). Outside the main lobe region, the CDF of the maximum sidelobe $r_{p}$, Equation (12), becomes

(13) $$F_{p}\left(r_{K}\right)={\left[F\left(r_{K}\right)\right]}^{N_{p}}={\left[1-e^{-10^{0.1\cdot K}}\right]}^{N_{p}}$$

where $N_{P}=int\left\{\frac{BT}{2}-1\right\}$.

For K=0, $F_{p}\left(r_{0}\right)={\left[1-e^{-1}\right]}^{N_{p}}≅0$ being $N_{p}\gg 1$ (BT≫1) and the probability that $r_{p}$ exceeds $-10\cdot log_{10}\left(BT\right)$ is very close to the unit.

Posing $F_{p}\left(r_{K}\right)=1-\delta$ and solving Equation (13), the corresponding value of K (in dB) is

(14) $$K=10\cdot log_{10}\left[-ln\left(1-\sqrt[{N_{p}}]{1-\delta }\right)\right]$$

**Figure 4:**
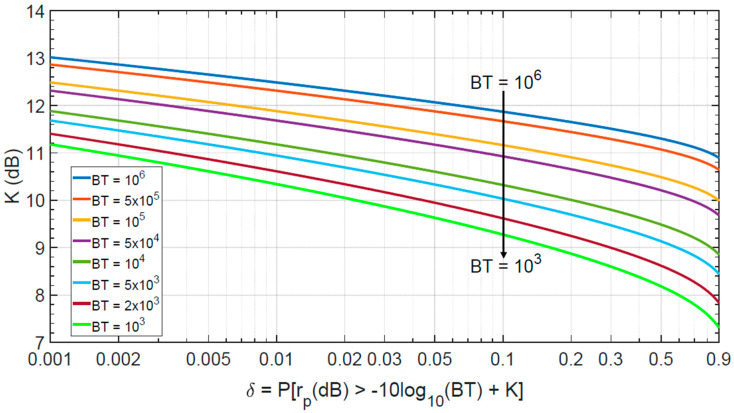
Parameter K (dB), as defined in Equation (2), versus δ with BT varying, Equation (14).

Figure 4 shows Equation (14) varying BT from $10^{3}$ to $10^{6}$ and δ from $10^{-3}$ to 0.9. Probability values δ in the order of 0.05 to 0.2 justify in Equation (2) the values of K in the range 10–12 dB to estimate the PSL for the, usually large, values of BT (order of $10^{4}–10^{6}$).

The sidelobes structure depends on the power spectral density of the generating noise. In the following it will be analyzed first in the exemplary case of uniform spectrum, then in the case of spectral shaping.

#### 2.2.1. Noise Waveforms with Uniform Power Spectrum

First, we consider a noise with bandwidth B and “uniform” power spectrum between $\left[-\frac{B}{2},+\frac{B}{2}\right]$:

(15) $$S_{0}\left(f\right)=\frac{1}{B}rect\left(f-\frac{B}{2}\right)$$

The variance (mean square frequency value) of $S_{0}\left(f\right)$ is $\frac{B^{2}}{12}$ and the corresponding ACF, equal to the correlation coefficient, is

(16) $$\rho_{0}\left(\tau \right)=\frac{\sin\left(\pi B\tau \right)}{\pi B\tau }$$

A “transition delay” $\tau^{*}$ may be defined equating the sidelobes envelope, given by the denominator of Equation (16), to the average random sidelobes level given by Equation (8):

(17) $$\eta_{r}=\sqrt{\frac{\pi }{4BT}}\dot{=}\frac{1}{\pi B\tau^{*}}$$

corresponding to $\tau^{*}=\sqrt{\frac{4T}{\pi^{3}B}}$. This delay $\tau^{*}$ divides the time axis in two intervals: the first $\left(0,\tau^{*}\right)$ in which the different realizations of the normalized ACF are very close to each other, in the main lobe and, possibly, in the first sidelobes; the second: $\tau >\tau^{*}$ (sidelobes region, after the transition zone), where the estimated mean value of the normalized ACF converges to the theoretical one, i.e., $\sqrt{\frac{\pi }{4BT}}$. In fact, for a single realization of the ACF, the sidelobes fluctuate according to the Rayleigh law, Equation (6).

Some computer simulation, shown in the following, highlight the above considerations. Let us consider an exemplary transmitted signal with Gaussian statistics, duration T=100 µs and rectangular power spectrum with B=50 MHz (BT=5000). In the sidelobes region, the theoretical mean value of r results, from Equation (8), $\eta_{r}=\sqrt{\frac{\pi }{4BT}}≅-38.04$ dB and $\tau^{*}B≅25.4$, i.e., there are 24 sidelobes out of the main lobe zone in time interval $\left(0,\tau^{*}\right)$.

**Figure 5:**
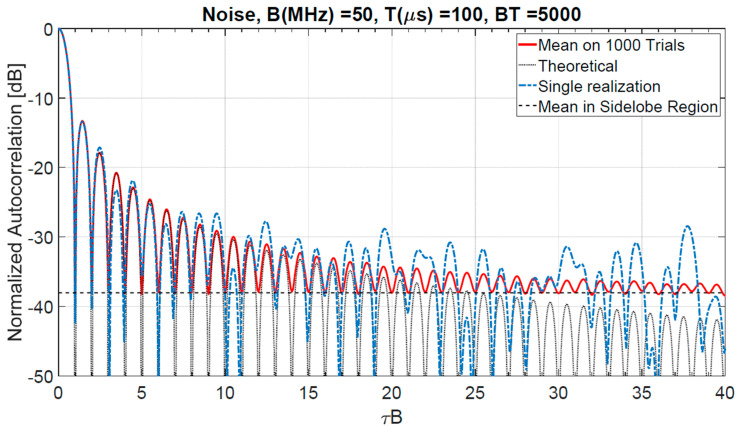
Normalized autocorrelation of a single realization: comparison between the single realization, the sample mean, and the theoretical value.

Figure 5 shows the result of a simulation of 1000 trials with a comparison between the correlation coefficient r of a single realization, the sample mean $\eta_{r}$ and the theoretical value of the ACF, Equation (16). The dashed horizontal line represents the theoretical mean value (−38.04 dB) in the sidelobes region. In the range 50<τB≤2500 of a simulated autocorrelation, a sample of 2450 realizations of r has been recorded. To get a quantitative evaluation of the simulative results, we have applied to this data-set the “Chi-squared-test” with a significance level α=0.05.

The “null” hypothesis:

$H_{0}$ ≝ {the random variable *r* is Rayleigh-distributed with PDF given by Equation (6)} is not rejected with a high *p*-value of 0.9.

In agreement with Equations (6) and (7), the probability density function of the *PSL* (i.e., of $r_{p}$) is:

(18) $$f_{p}\left(r\right)=N_{p}\cdot {\left[F\left(r\right)\right]}^{N_{p}-1}\cdot f\left(r\right)=N_{p}\cdot {\left[1-e^{-\frac{r^{2}}{4BT}}\right]}^{N_{p}-1}\frac{r}{2BT}e^{-\frac{r^{2}}{4BT}}\cdot U\left(r\right)$$

By simulation on 10,000 trials with $N_{p}=2000$, applying the Chi-squared-test with α=0.05, the null hypothesis:

$H_{0}$ ≝ {the random variable $r_{p}$ is distributed with PDF given by Equation (18)} is not rejected with a high *p-*value *of* 0.6.

#### 2.2.2. Noise Waveforms with “Shaped” Power Spectrum

In most NRT applications the time delays τ of interest respect the inequality τ≪T, and the “uniform” spectral density $S_{0}\left(f\right)$ in Equation (15) may be made more convenient in the region of interest using an “*m*-order convolution” to shape the spectrum in order to reduce the PSL:

(19) $$S_{m}^{WB}\left(f\right)=\left\{\frac{1}{B}rect\left(f-\frac{B}{2}\right)\right\}{⨂}_{m}\left\{\frac{1}{B}rect\left(f-\frac{B}{2}\right)\right\}$$

leading to normalized ACF:

(20) $$\rho_{m}^{WB}\left(\tau \right)={\left[\frac{\sin\left(\pi B\tau \right)}{\pi B\tau }\right]}^{m+1}$$

This generalization, hereafter named “WB” for wideband, assumes an unlimited availability of spectrum. In fact, with m increasing, the power spectrum tends to a Gaussian shape with an increasing variance equal to $\frac{1}{12}\left(m+1\right)B^{2}$, i.e., to a wideband noise, corresponding to a narrower and narrower main lobe of the autocorrelation function.

Hence, the following evaluations consider a more realistic generalization named “NV” (normalized-variance) reduces the spectrum variance (back to the “original” value $\frac{B^{2}}{12}$) applying to the frequency axis the scaling factor:

(21) $$\alpha_{m}={\left(m+1\right)}^{-1/2}$$

The resulting spectrum and the corresponding normalized ACF are

(22) $$S_{m}^{NV}\left(f\right)=\;\frac{1}{\alpha_{m\;}}S_{m}^{WB}\left(\frac{f}{\alpha_{m\;}}\right)$$

(23) $$\rho_{m}^{NV}\left(\tau \right)={\left[\frac{\sin\left(\pi B\alpha_{m}\tau \right)}{\pi B\alpha_{m}\tau }\right]}^{m+1}$$

**Figure 6:**
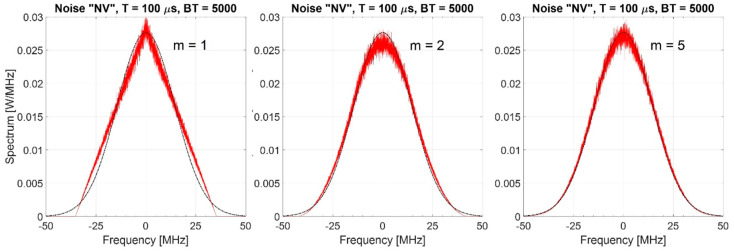
Spectrum of the “Normalized Variance”, i.e. “NV”, noise waveform (estimated on 1000 trials), BT = 5000. Dashed-line shows the Gaussian spectrum, *m* = 1, 2, 5.

In Table 2 the normalized bandwidths (wrt B) at −3, −6, −10, −20, and −40 dB are shown for m=1, 2, 3, 4, 5, and 10. They are evaluated by applying Equations (19) and (22). We observe that for m≥2 the bandwidth $B_{-6}≅B$, highlighted in bold face in Table 2. Figure 6 shows, for m=1, 2, and 5 by simulation of 1000 trials, the mean values of the spectrum.

**Figure 7:**
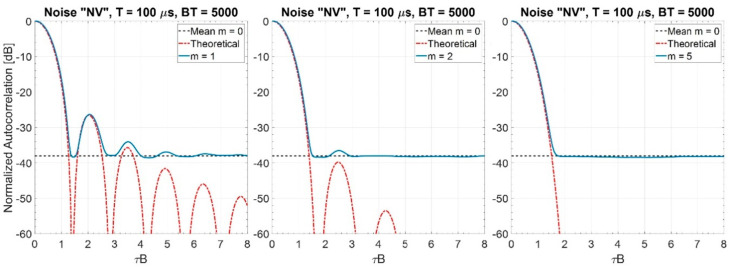
Normalized autocorrelation of “NV” noise waveforms for BT = 5000 (values are estimated on 1000 trials), *m* = 1, 2, and 5.

As one could expect because of the “central limit theorem”, for m equal to (or greater than) some quite low value (e.g., for m≥5), the spectrum is very similar to the Gaussian one with variance $\frac{B^{2}}{12}$. Figure 7 shows the corresponding mean values of the normalized autocorrelation. We note that

(i) the main lobe width is ≅1/B; and
(ii) the mean value of the PSL is $\eta_{r}≅\sqrt{\frac{\pi }{4B_{6}T}}$(regardless of m), a value very close to $\sqrt{\frac{\pi }{4BT}}$ corresponding to m=0.

A third generalization of the spectrum considers situations in which the spectral occupancy is “severely-controlled” (hereafter named “SC”), i.e., the whole PSD has to strictly stay in the $\left[-\frac{B}{2},\;+\frac{B}{2}\right]$ interval. In this case the scaling factor, corresponding to the one of Equation (21), becomes

(24) $$\beta_{m\;}={\left(m+1\right)}^{-1}$$

The resulting spectrum and the corresponding normalized ACF are

(25) $$S_{m}^{SC}\left(f\right)=\;\frac{1}{\beta_{m\;}}S_{m}^{WB}\left(\frac{f}{\beta_{m\;}}\right)$$

(26) $$\rho_{m}^{SC}\left(\tau \right)={\left[\frac{\sin\left(\pi B\beta_{m\;}\tau \right)}{\pi B\beta_{m\;}\tau }\right]}^{m+1}$$

**Figure 8:**
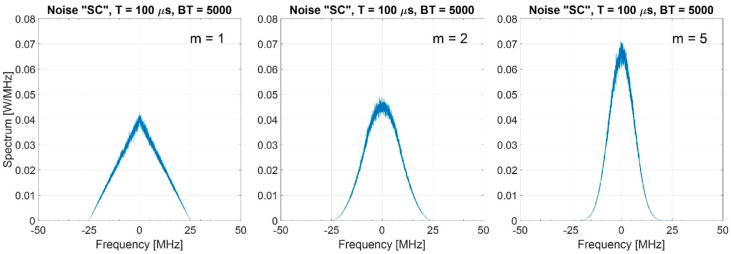
Spectrum of the “SC” noise waveforms (estimated on 1000 trials), BT = 5000, m=1, 2, 5.

**Figure 9:**
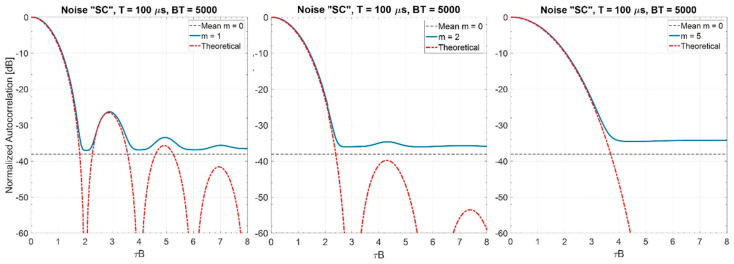
Normalized autocorrelation of “SC” noise waveforms, BT = 5000, *m* = 1, 2, and 5 (values are estimated on 1000 trials).

Table 3 reports the normalized bandwidths. Increasing m, the bandwidth is severely reduced (Figure 8) and the main lobe-width and the mean values of the normalized ACF in the sidelobes region increase as shown in Figure 9.

Very preliminary experimental results using noise waveform “NV” with *m* = 1, 2, and 5 are published in [18].

**Figure 10:**
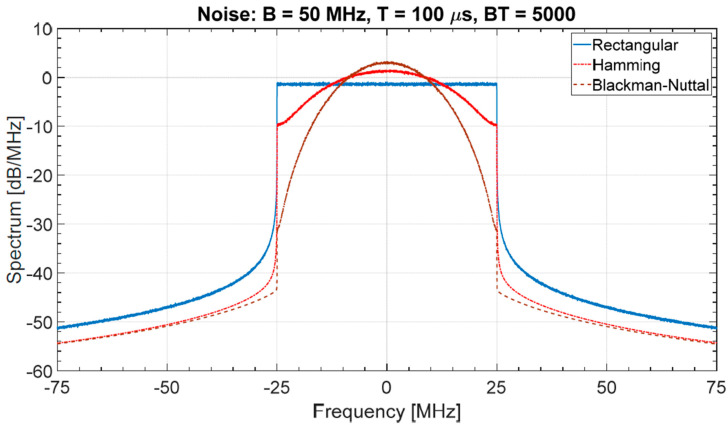
Shaped spectrum of a noise spectrum by Rectangular, Hamming, and Blackman-Nuttall window (values are estimated on 1000 trials).

A classical approach to strictly control the bandwidth uses spectral windows [19] to suitably shape the spectrum in order to have a low level of the sidelobes of the related ACF. Figure 10 shows the mean spectrum (simulated on 1000 trials) of a noise waveform shaped by three different spectral windows: Rectangular, Hamming, and Blackman-Nuttall.

**Figure 11:**
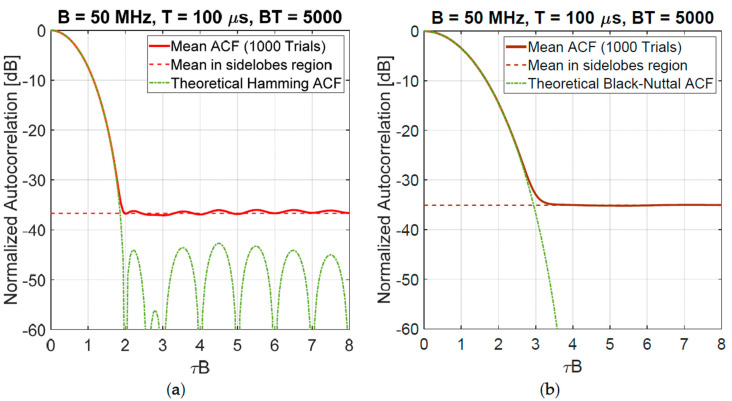
Normalized autocorrelation function (ACF): (**a**) Hamming noise waveform ${\hat{\eta }}_{r}=36.69$ dB; (**b**) Blackman-Nuttall noise waveform ${\hat{\eta }}_{r}=35.08$ dB. Case (**a**) grants a better range resolution.

Theoretically, the Blackman-Nuttall spectral weighting applied to deterministic waveforms permits to reach very low sidelobe levels (<−100 dB) in the normalized ACF. However, such an excellent performance is not achieved with noise waveforms because of the “random fluctuations” of the sidelobes as discussed above. An example is shown in Figure 11, where the mean normalized ACF (evaluated with 1000 Monte Carlo trials) is compared with the theoretical one. The constant level (dashed line in Figure 11) corresponds to the estimated mean value (sample mean) in the sidelobes region, i.e., ${\hat{\eta }}_{r}$. Inverting Equation (8) we may define an equivalent band: $B_{eq}=\frac{\pi }{4{\hat{\eta }}_{r}^{2}T}<B$ for a noise signal, corresponding to a waveform with uniform spectrum and sidelobe levels of the ACF Rayleigh-distributed with parameter $a_{Ray}^{2}=\frac{1}{2B_{eq}T}$. By simulation, applying the Chi-squared-test with α=0.05, the null hypothesis: $H_{0}$ ≝ {the random variable *r* is Rayleigh-distributed} is not rejected with *p*-value = 0.90 and 0.42 for Hamming and Blackman-Nuttall, respectively. This example shows that the “random fluctuations” of sidelobes in NRT may lead to prefer “less performing” windows, unless sidelobes suppression methods “are applied to each generated waveform”, as explained in Section 3.

#### 2.2.3. The Range Transition Zone

In the previous analysis, it has been shown that the ACF has two main intervals: one, around zero delay, in which the deterministic shape, related to the PSD, dominates, and another, at larger delays, where random sidelobes dominate with a level depending on the *BT* product. A transition zone is in between. Considering the cases analysed in Section 2.2.2, for the “*NV*” case the bandwidth and the mean PSL show a weak dependence on the parameter m. Hence, similar to Equation (17) it is possible to define a “transition lag zone” with central value $\tau^{*}$ and width $\frac{1}{B}$ (the nominal duration of a sidelobe of the ACF), after which the peaks of the “nominal” autocorrelation function are embedded in the random fluctuations.

The value of $\tau^{*}$ is obtained equating the denominator of Equation (23) with the mean value $\eta_{r}$:

(27) $${\left(\frac{1}{\pi B\alpha_{m}\tau^{*}}\right)}^{m+1}=\sqrt{\frac{\pi }{4}\cdot \frac{1}{B\cdot T}}$$

Normalizing to the waveform duration T, i.e., using $\gamma =\frac{\tau^{*}}{T}$:

(28) $$\gamma ={\left(\frac{2}{\pi }\right)}^{2}\sqrt{m+1}{\left(\frac{4}{\pi }BT\right)}^{-\frac{2m+1}{2m+2}}$$

In the ordinary condition of BT≫1, from the above formulas and from simulation it results that the delay interval in which the “true” autocorrelation function dominates is a very small fraction of T.

**Figure 12:**
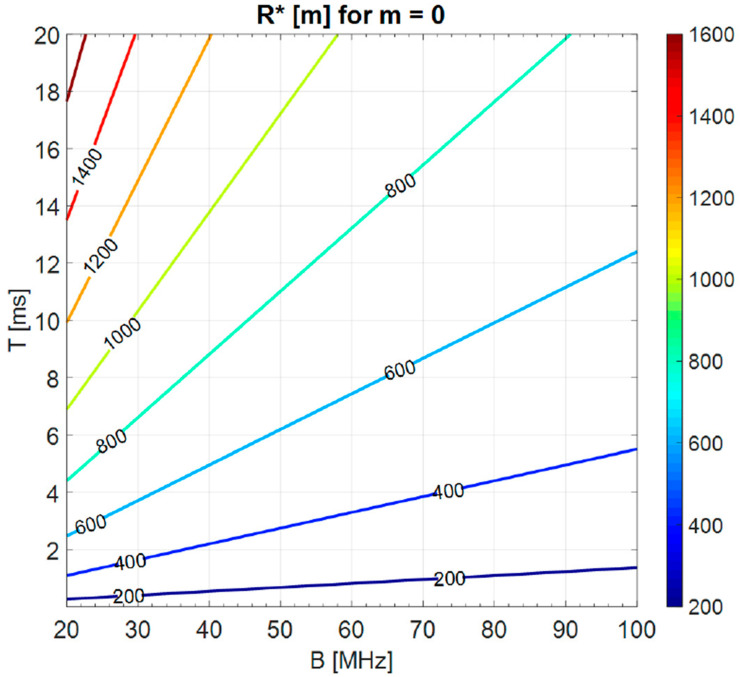
Level curves (in meters) of the transition Range R* varying B and T in the ideal case of flat spectrum (*m* = 0).

In the ideal case of a perfectly flat spectrum constrained in the considered bandwidth B (i.e., m=0), typical values of the “transition range” $R^{*}=\frac{c}{2}\tau^{*}$, varying B (20–100 MHz) and T (0.2–20 ms), are of the order of hundreds of meters with a maximum around 1.6 km (see Figure 12). The corresponding autocorrelation (see for instance Figure 5) has the highest sidelobes, with a maximum −13.2 dB level below the main peak.

**Figure 13:**
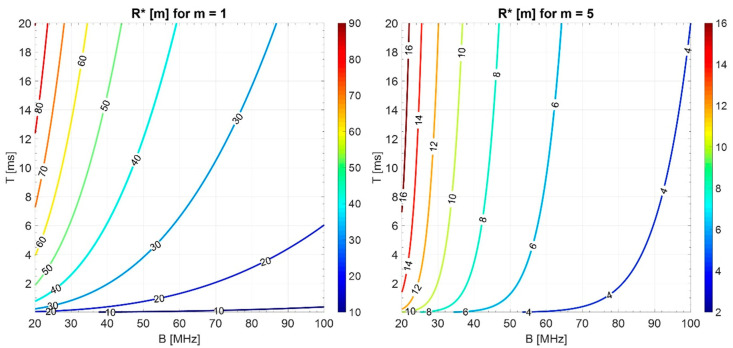
Level curves (in meters) of the transition Range R* for “NV” varying B and T, for *m* = 1 and *m* = 5.

In the practical case of lowering these “deterministic” sidelobes by frequency-domain weighting, i.e., smoothing the spectrum (m≥1), $R^{*}$ would be reduced. Some exemplary values of $R^{*}$ for m=1 and m=5 are shown in Figure 13.

With m=1, the transition region is of the order of tens of meters, while for m=5 it is of the order of meters. Note that these are the typical delay intervals of the above-discussed critical phenomena, i.e., the antenna leakage (Section 1) and the close-in clutter. To cope with this “short range” problem, a method to suppress the sidelobes of the autocorrelation function in a limited delay interval is introduced in the next section.

Summing up, to mitigate the sidelobes when a relatively large BT is needed (order of hundred of thousand or more) the most practical way seems to act not only on the “random process” (i.e., the overall set of waveforms) but also on its “realizations” (i.e., on each waveform), paying the price of a potential reduction of LPI/LPE. The topic of Range sidelobe attenuation has been widely published in the literature for both deterministic radar signals [1,2] and noise radar signals [15,16,20,21,22,23]. A computationally simple, effective method, suited to CE noise radars, is presented in Section 3.

## 3. Range Sidelobes Suppression for the Autocorrelation Function of Noise Waveforms

### 3.1. The FMeth (Filtering Method) Algorithm: General Description

**Figure 14:**
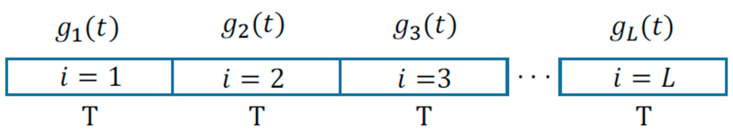
Coherent intervals in emission.

We consider continuous emission noise radar for the rationales described in the Introduction. The emission has coherent time intervals with duration T (Figure 14), with $T\leq T_{D}$, where $T_{D}$ is equal to or smaller than the decorrelation time (or the dwell time).

For each i, $g_{i}\left(t\right)$ (*i* = 1, 2, …, *L*) defines a different realization of a band-limited (within *B*) random process. In almost all practical cases, the radar designer sets $T\gg T_{max}$, defined in term of maximum range $R_{max}=\frac{cT_{max}}{2}$, in order to obtain a large enough processing gain BT.

**Figure 15:**
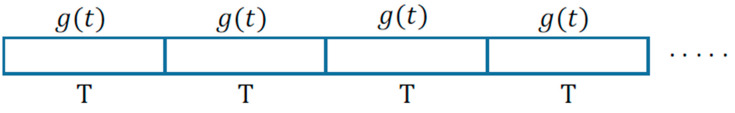
Periodic signal in emission.

In the following we use the scheme of Figure 15, creating a periodic signal structure by a single realization g(t), i.e., a circular convolution in the ACF computation. With $T\gg T_{max}$ and BT≫1 the approximation of the ACF and of the spectrum of g(t) with their sampled and periodical versions are quite good. We assume a sampling interval equal to $\frac{1}{k_{F}\cdot B}$ with an oversampling factor $k_{F}>1$.

The signal g(t), realization (sample function) of the random noise process, is generated starting from the design power spectral density (PSD) of the process, i.e., $E\left[{\left|G\left(f\right)\right|}^{2}\right]$, being G(f) the Fourier transform of g(t). The ACF of g(t), i.e., the output of the compression filter (matched filter), is (hereafter we use the continuous-time non-causal formalism for simplicity):

(29) $$R\left(\tau \right)=g\left(\tau \right)⨂g^{*}\left(-\tau \right)$$

where ⨂ is the convolution operator. The Fourier transform of Equation (29) is

(30) $$S\left(f\right)=G\left(f\right)\cdot G^{*}\left(f\right)={\left|G\left(f\right)\right|}^{2}$$

**Figure 16:**
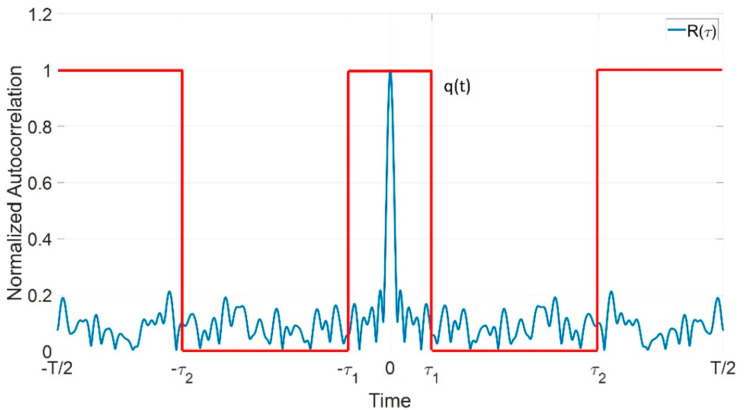
The sidelobes suppression interval as defined by q(τ), red line.

Let $\tilde{R}\left(\tau \right)=R\left(\tau \right)\cdot q\left(\tau \right)$ be the desired autocorrelation obtained by multiplying R(τ) by

(31) $$q\left(\tau \right)=\{\begin{array}{l} 0\;\;\;\left[-\tau_{2}<\tau <-\tau_{1}\right]⋃\left[\tau_{1}<\tau <\tau_{2}\right]\;\\ 1\;\;\;\;\;\;\;\;\;\;\;\;elsewhere\; \end{array}$$

in order to suppress the range sidelobes inside the time interval $\left(\tau_{1},\tau_{2}\right)$, as described in Figure 16.

The product R(τ)·q(τ) corresponds, in the frequency domain, to the convolution of the spectrum S(f) with the Fourier transform of q(τ). Denoting with ℱ{·} the Fourier transform operator, it results in

(32) $$\tilde{S}\left(f\right)=ℱ\left\{\tilde{R}\left(\tau \right)\right\}=S\left(f\right)⨂ℱ\left\{q\left(\tau \right)\right\}$$

For how q(τ) is defined, the ℱ{q(τ)} contains spectral components outside the band (i.e., −B/2 to +B/2) of g(t). Using the FFT to implement the Fourier transform, we should then “accept” the harmful effects introduced by the folding of the spectral components outside the band B, as shown in the ensuing results.

Let H(f) be the frequency response of a filter such that

(33) $$\tilde{S}\left(f\right)={\left|H\left(f\right)\right|}^{2}\cdot S\left(f\right)$$

Hence, from Equations (30) and (33), the frequency response of this sidelobes suppression filter is

(34) $$H\left(f\right)=\frac{\sqrt{\tilde{S}\left(f\right)}}{\left|G\left(f\right)\right|}$$

**Figure 17:**
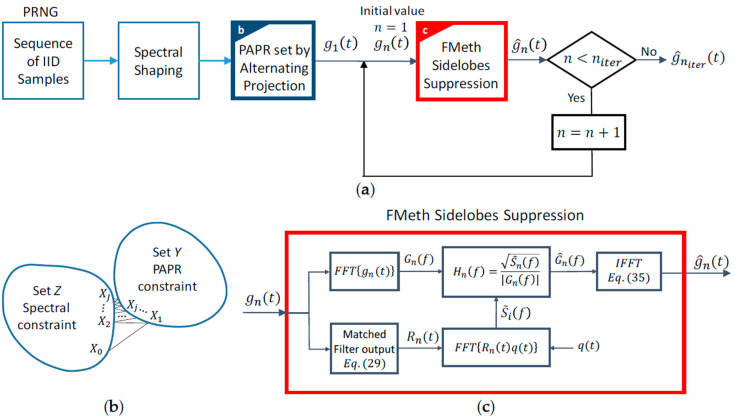
(**a**) Functional block diagram of the generation of a noise waveform. (**b**) The “Alternating Projection” method. (**c**) Functional block diagram of the FMeth algorithm to suppress the sidelobes.

This approach, here named FMeth and based on the concept of “inverse filtering”, is well known in literature (see, for instance, [20]). The systematic description of the noise waveforms generation with sidelobes suppression by FMeth is shown in Figure 17a.

#### 3.1.1. PRNG and Spectral Shaping

Starting from a pseudo-random number generator (PRNG), a sequence of independent and identically distributed (i.i.d.) Gaussian complex samples with assigned power spectrum (spectral shaping) of a band-limited B, is generated by filtering, see Section 2.2.2.

#### 3.1.2. PAPR Setting by Alternating Projection

After spectral shaping, the PAPR of the waveform is around 10−12 or greater (see the Introduction). To reduce the loss due to high PAPR (Table 1), the PAPR can be forced to a lower value (e.g., 1.5−2.0) using the “alternating projection” algorithm [24], which is a powerful and computationally efficient way to design waveforms with a given structural properties (e.g., prescribed norm, PAPR, etc. …). More specifically, the alternating projection method efficiently applies to the huge class of Inverse Eigenvalues Problems (IEP) aimed at creating a structured waveform or a structured “tight frame”. A tight frame is a generalization of an orthogonal system [25] and, according to [24], is described by its spectrum. Because of the spectral characterization of a tight frame, IEP is the most suitable solution for seeking the structured frame with prescribed spectral properties. The idea behind the alternating projection algorithm is to search for a point of intersection between a matrix that satisfies a given spectral constraint and a matrix that satisfies a given structural constraint. In our case, the spectral constraint (matrix *Z*) is determined by the spectral occupancy of the generated waveform and the structural constraint (matrix Y) is the desired PAPR. Hereafter we refer to a matrix (and not to a single waveform vector) since the IEP, of which alternating projection algorithm is an efficient solver, operates on tight frame [25]. Figure 17b depicts the alternating projection method in which two sets of matrixes Z (e.g., spectral constraint) and Y (e.g., structural constraint) are iteratively projected onto each other in order to find the intersection matrix X that satisfies both constraints. The subscript “j” represents the number of iterations and the initial matrix $X_{0}$ represents the starting noisy band-limited sequence. The algorithm details can be found in Appendix B.

#### 3.1.3. FMeth Sidelobes Suppression

The functional block diagram of the FMeth algorithm is shown in Figure 17c. The step-by-step description is summarized as follows.

- The sample autocorrelation R(τ) of the PAPR-regulated sequence is computed.
- Zeroing the points of R(τ) between $\tau_{1}$ and $\tau_{2}$ by multiplication with q(τ), Equation (31), the desired autocorrelation $\tilde{R}\left(\tau \right)=R\left(\tau \right)\cdot q\left(\tau \right)$ is determined.
- The spectrum $\tilde{S}\left(f\right)$ and the Fourier transform G(f) of g(t) are estimated by FFT, and, from Equation (34), the frequency response H(f) is evaluated.
- The random signal $\hat{g}\left(t\right)$, whose autocorrelation function shows attenuated sidelobes inside the time interval $\left(\tau_{1},\tau_{2}\right)$, is generated by the following procedure:

(35) $$\hat{g}\left(t\right)={ℱ}^{-1}\left\{\hat{G}\left(f\right)\right\}={ℱ}^{-1}\left\{H\left(f\right)\cdot G\left(f\right)\right\}$$

Due to the detrimental effects related to the use of the FFT, i.e., the folding of the spectral components of q(t) into (−B/2, +B/2), the output $\hat{g}\left(t\right)$ has to be iteratively processed by the FMeth algorithm for a suitable number of times ($n_{iter}$) to improve the suppression sidelobes, as shown in Figure 17a.

At the end of the suppression procedure, i.e., after a number $n_{iter}$ of iterations suited to satisfy the requirement of the PSL, the “tailored” sequence ${\hat{g}}_{n_{iter}}\left(t\right)$ can be used as transmitted waveform. We underline that a possibly large number of iterations is not a practical problem being this procedure designed for an offline-preparation of a large number of transmit signals.

### 3.2. Results for Zero Doppler–Sidelobes Level in the ACF

#### 3.2.1. Sensitivity of the Sidelobe Attenuation to the Number of Iterations

**Figure 18:**
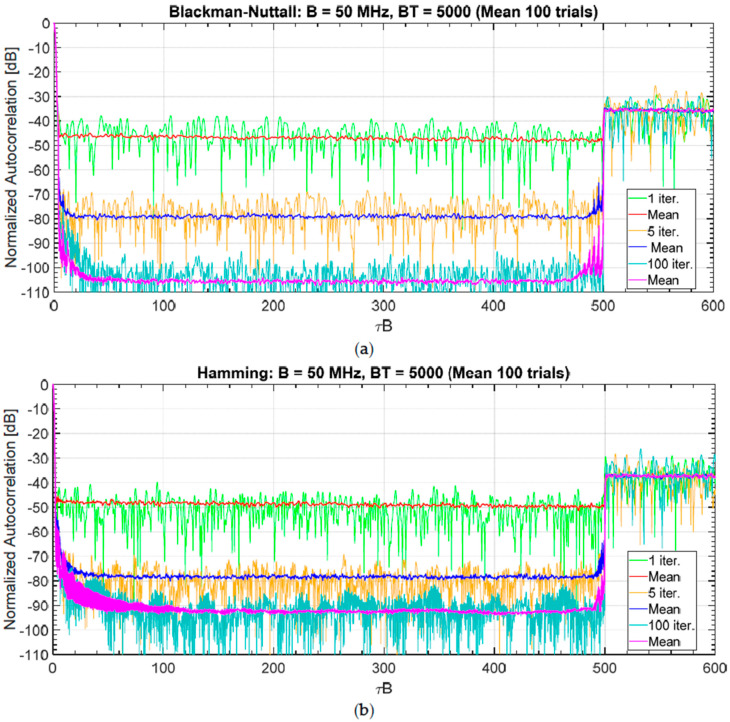
Normalized ACF when the FMeth is used to suppress a sidelobes region (varying the number of iterations: *n_iter_* = 1, 5, 100) for a single realization and averaging 100 trials. The PAPR assumes its “naturally” value (10–12). The suppression zone is the 20% of the total. (**a**) Blackman-Nuttall spectrum, and (**b**) Hamming spectrum.

To analyse the effect related to the number of iterations in the FMeth algorithm, some exemplary results are obtained starting from a noise waveform, i.e., a realization of a complex Gaussian process with both a Blackman-Nuttall and a Hamming spectrum setting B=50 MHz, T=100 μs (BT=5000), sampling frequency $F_{s}=16\cdot B$. To analyse the dependence of FMeth on the number of iterations ($n_{iter}$), no constraint is applied to the PAPR (which “naturally” takes values around 10−12 or greater) and the percentage of the suppression zone is set in the interval $\tau_{1}B=4,\;\tau_{2}B=500$ (in range $R_{1}=12$ m, $R_{2}=1500$ m) corresponding approximately to the 20% of the total duration. The results concerning the sidelobes attenuation are obtained for three values of $n_{iter}:1,\;5,\;100$. Figure 18a,b shows the results for Blackman-Nuttall and Hamming noise waveforms, respectively. The sidelobes suppression is poor for a single iteration (the improvement is only 12 dB wrt the mean PSL). With five iterations, the PSL reaches (−80, −78 dB) in mean and, for $n_{iter}=100$, the PSL decreases up to (−95, −80 dB) for a single realization and (−105, −93 dB) for the mean.

**Figure 19:**
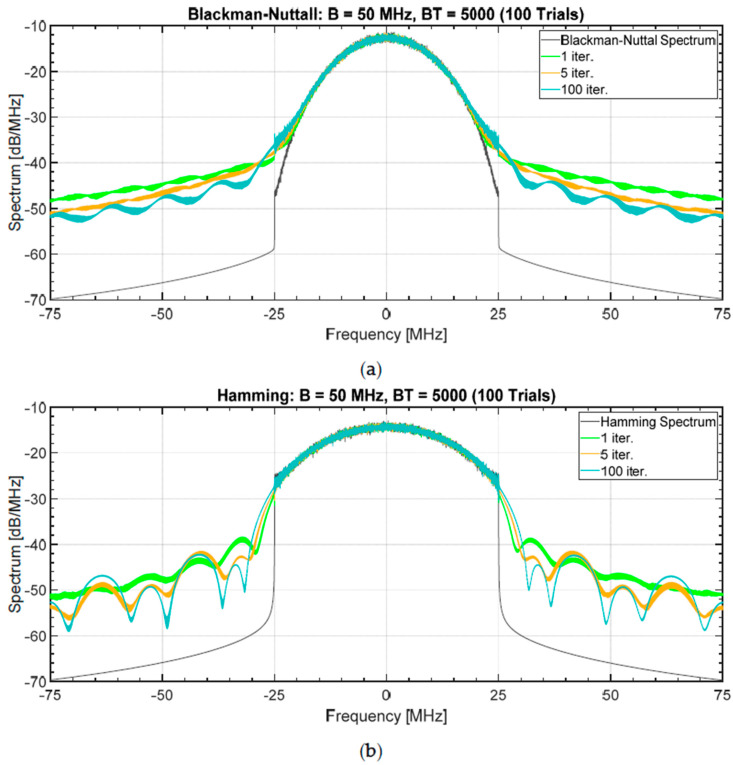
Mean spectrum (FMeth, 100 trials) varying the number of iterations: *n_iter_* = 1, 5, and 100. The PAPR assumes its “natural” value in the range 10–12. The suppression zone is the 20% of the total. (**a**) Blackman-Nuttall spectrum, and (**b**) Hamming spectrum.

Figure 19a,b shows the corresponding mean spectrum as compared with the original sequence. It is interesting to notice that both the distortion introduced by the non-linearity and the folding of the spectral components outside the band B look acceptable.

#### 3.2.2. Sensitivity of the Sidelobe Attenuation to the PAPR and to the Width of the Suppression Zone

The results shown in Section 3.2.1 are obtained for a Gaussian noise in the case of a linear system with full dynamic range (no constraint on the PAPR) and supposing a 20% of suppression zone with respect to the total range. If the PAPR is varied (i.e., decreased up to one) and the suppression zone is increased (up to 100%), the effectiveness of the FMeth algorithm is significantly reduced.

To quantify these effects, for BT=5000 and $n_{iter}=100$, we have set different PAPR values (9.0, 5.0, 2.0, 1.5, 1.0) and percentages of the suppression zone (20, 40, 60, 80, 95, and 100 per cent).

The “natural” PAPR of the starting noise waveform, i.e., $g_{1}\left(t\right)$ in Figure 17a, can be forced to a different (lower) value (see Figure 17b). Being the FMeth generation based on a “mild” filtering, which suppresses spectral components whose energy (residing in the sidelobes of the ACF) is a small fraction of total energy (mainly allocated in the main lobe of the ACF), one could guess that this sidelobes suppression does not significantly modify the PAPR as initially imposed, even when the suppression zone increases. Simulations have confirmed this hypothesis.

Table 4 (for the Blackman-Nuttall spectrum) and Table 5 (for the Hamming spectrum) define a matrix whose columns (for a given PAPR set) report the mean PSL varying the percentage of the suppression zone, while the rows (for a percentage set) show the mean PSL varying the PAPR.

These values can be explained by the following considerations. First, decreasing the PAPR the number of degrees of freedom is reduced (the amplitude variation is constrained). Second, a larger suppression zone, maintaining fixed the number of degrees of freedom, makes the suppression algorithm less efficient, being applied over a wider range. From the results, we can conclude that a good trade-off can be reached with, the Hamming spectrum, a suppression zone of the 20%, and a PAPR equal to 1.5, whose loss is still acceptable (see for the loss Table 1).

### 3.3. Doppler Sensitivity and Sidelobes Suppression for the Ambiguity Function

In the presence of moving targets, the Doppler effect causes the well-known mismatching in the compression filter. The frequency shift produces two effects: (i) loss on the peak at the output of the matched filter (i.e., loss in SNR); and (ii) reduced effectiveness of the sidelobes suppression algorithm.

**Figure 20:**
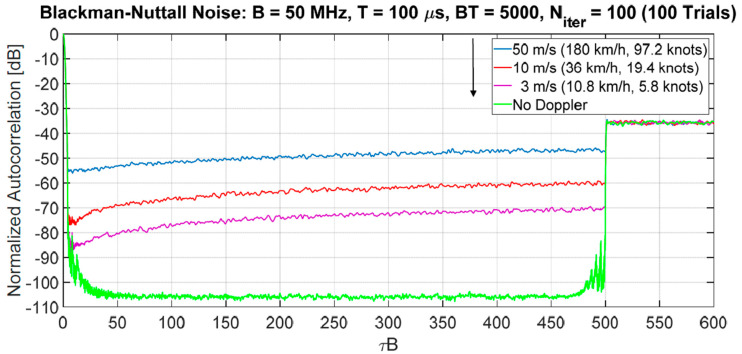
Normalized ACF with suppression sidelobes varying the Doppler velocity 3, 10, and 50 m/s. BT = 5000, *n_iter_* = 100. Mean on 100 trials.

At X-band (λ=0.032 m) for noise waveforms generated starting from the Blackman-Nuttall spectrum (B=50 MHz, BT=5000), we have considered three values of the Doppler velocity: $v_{D}=3,\;10,\;50$ m/s. In Figure 20, the normalized ACFs are shown varying $v_{D}$ and compared with the zero Doppler case. No constraint is imposed on the PAPR and the suppression zone is set to 20% of the total duration. By simulation, for $v_{D}\leq 20$ m/s the loss in SNR is negligible (<0.1 dB), increasing $v_{D}$ up to 50 m/s the loss is still acceptable, i.e., −0.59 dB. The suppression algorithm was applied imposing 100 iterations.

The range sidelobes suppression algorithm here introduced, as shown, is fairly sensitive to the Doppler shift. Hence, two solutions are possible. The first, and “classical”, one tries to “tailor” noise waveforms to yield low sidelobes of their “ambiguity function”, i.e., both in “range” (time) and in “Doppler” (frequency). Methods to resolve this problem are described in [21,22]. The second, novel solution, most effective from the application point of view, uses the “Range filters bank” (RFB) as described in [26,27]. This solution has a “fast time” processing of sequences of duration $T_{r}$, which is very slightly affected by the Doppler thanks to the choice of $T_{r}$, being $T_{r}\ll T$, with a downstream “slow time” processing generating a bank of Doppler filters to cover the whole velocity interval of interest, in a way similar to the well-known moving target detector (MTD) [28,29].

## 4. Comments, Conclusions, and Short Reference to Future Experimental Work

By definition, the transmitted waveforms form the key element of the NRT and the driver of its main advantages as described in this introductory paper. These advantages are best exploited in the continuous emission configuration, which is the one considered here.

However, the simultaneous transmission and reception of a CE radar puts demanding requirements both to the radar hardware, mainly in terms of the leakage from the transmitting antennas to the receiving one, and to the radar waveforms. The latter have very demanding requirements on Range and Doppler sidelobes, on energetic effectiveness (PAPR) and on compatibility with the regulation for the use of the electromagnetic spectrum (see Appendix A). For military radars, more requirements add in terms of robustness against “interception” and “exploitation” of the radiated signals by an adversary part.

Hence, a novel design procedure for CE noise radar waveforms has been discussed with the related analytical and simulative results.

Ensuing papers of this special issue will tackle other facets of the NRT waveforms and present more results. In particular, a second paper will be submitted by the same team to treat in more practical way the results of the waveforms design as checked by trials in different operating environments with the pertaining lessons learnt, while a third planned paper will deal with the counter-interception and counter-exploitation features and the related analysis methods based on the information theory.

## Figures and Tables

**Table 1:** Signal-to-noise ratio (SNR) loss (dB) versus peak-to-average power ratio (PAPR).

PAPR	Loss (dB)
1.0	0.0
1.25	0.97
1.5	1.76
1.75	2.43
2.0	3.01
3.0	4.77
5.0	6.99
7.0	8.45
10.0	10.0

**Table 2:** Normalized bandwidth (−3, −6, −10, −20, and −40 dB) of the spectrum for “NV” waveforms.

“NV” Waveforms					
m	B_−3_/B	B_−6_/B	B_−10_/B	B_−20_/B	B_−40_/B
**1**	0.67	**1.06**	1.27	1.34	1.41
**2**	0.73	**1.02**	1.28	1.42	1.72
**3**	0.72	**1.00**	1.26	1.42	1.93
**4**	0.70	**0.98**	1.25	1.68	2.06
**5**	0.71	**0.99**	1.24	1.41	2.15
**10**	0.69	**0.98**	1.25	1.41	2.32

**Table 3:** Normalized bandwidth (−3, −6, −10, and −20 dB) of the spectrum for “SC” waveforms.

“SC” Waveforms				
m	B_−3_/B	B_−6_/B	B_−10_/B	B_−20_/B
**1**	0.50	**0.75**	0.90	0.95
**2**	0.42	**0.59**	0.74	0.82
**3**	0.36	**0.50**	0.63	0.71
**5**	0.29	**0.40**	0.51	0.58
**10**	0.21	**0.29**	0.38	0.43

**Table 4:** Mean peak side lobe (PSL) (dB) on 100 trials using FMeth varying the PAPR and the percentage of the suppression zone. Noise waveforms with Blackman-Nuttall spectrum.

Blackman-Nuttall: B = 50 MHz, T = 100 μs, BT = 5000					
Suppression Zone [%]	PAPR 9.0	PAPR 5.0	PAPR 2.0	PAPR 1.5	PAPR 1.0
20	−103.9	−100.8	−89.3	−75.9	−57.9
40	−104.3	−101.1	−79.6	−66.3	−54.5
60	−102.5	−95.2	−71.1	−60.7	−52.3
80	−99.7	−88.6	−63.0	−56.9	−51.5
95	−89.1	−78.3	−58.8	−55.1	−50.6
100	−83.1	−69.5	−56.5	−53.8	−49.9

**Table 5:** Mean PSL (dB) on 100 trials using FMeth varying the PAPR and the percentage of the suppression zone. Noise waveforms with Hamming spectrum.

Hamming: B = 50 MHz, T = 100 μs, BT = 5000					
Suppression Zone [%]	PAPR 9.0	PAPR 5.0	PAPR 2.0	PAPR 1.5	PAPR 1.0
20	−92.3	−93.6	−89.9	−76.8	−59.3
40	−90.7	−93.3	−80.3	−67.5	−56.0
60	−88.4	−89.1	−71.7	−61.9	−54.0
80	−84.0	−82.9	−64.3	−58.3	−52.9
95	−79.2	−74.9	−60.2	−56.4	−52.2
100	−73.1	−68.3	−58.2	−55.0	−51.4

**Figure A1:**
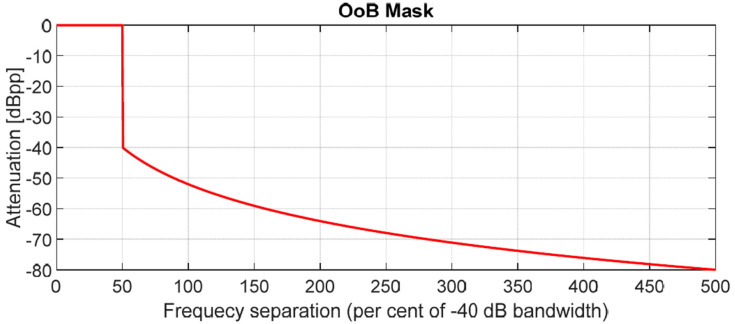
Design objective for radar systems.

**Figure A2:**
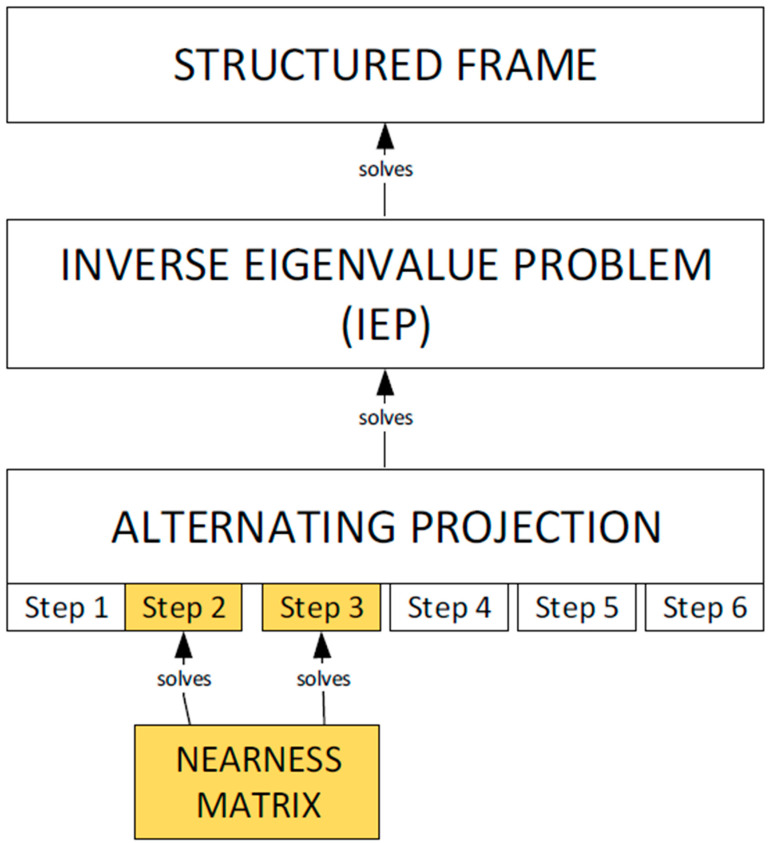
Solution to the Structured Frame problem in turn addressed by Alternating Projection and Nearness Matrix problem.

## Appendix A. Band Occupancy in NRT Applications

The *m-*order convolution, defined by Equation (19), and the “frequency-scaling” factor of Equation (22), allow to dispose of a very simple mathematical closed-form model for the spectrum of noise signals, with a good approximation of the Gaussian PSD when m is above the order of a very few units. Increasing m, the −40 dB bandwidth increases (see Table 2) and for m=4 the bandwidth $B_{-40}≅2B$. For radar applications, ITU-R recommendations [9] on the unwanted emissions in the “out-of-band” (OoB) domain (i.e., the adjacent frequency region employed by other applications) define the limits of the mask for the allocated band, the OoB and the spurious emission. The most recent and tight recommendations assert that radar systems should be designed to meet the requirement of the mask-roll-off at 40 dB per decade from the $B_{-40}$ dB bandwidth to the spurious level (Figure A1), as shown in Figure 18.

## Appendix B. The “Alternating Projection” Algorithm

The idea behind the alternating projection algorithm is to search for a point of intersection between a matrix describing a spectral constraint and a matrix describing a structural constraint. In our case, the spectral constraint is determined by the spectral occupancy of the generated waveform and the structural constraint is the desired PAPR.

In [24], the alternating projection algorithm is well described. Here we report the main steps for the sake of simplicity.

Let us suppose we have two set of matrices Y and Z of the same dimension, with the former implementing spectral constraint and the latter implementing the structural constraint. The alternating projection algorithm is described as follows:

INPUT

- An (arbitrary) initial matrix $Y_{0}$
- The number of the iteration, J

OUTPUT

- A matrix $\bar{Y}$ in Y and a matrix $\bar{Z}$ in Z

PROCEDURE

(1) Initialize j=0
(2) Find a matrix $Z_{j}$ in Z such that
  $$Z_{j}\in arg\underset{Z\;\in \;Z}{\min}∥Z-Y_{j}∥{}_{F}$$
  being ${∥\cdot ∥}_{F}$ operator the Frobenius norm.
(3) Find a matrix $Y_{j+1}$ in Y such that
  $$Y_{j+1}\in arg\underset{Y\;\in \;Y}{\min}∥Y-Z_{j}∥{}_{F}$$
(4) Increment j by one.
(5) Repeat steps 2–4 until j=J
(6) Let $\bar{Y}=Y_{J}$ and $\bar{Z}=Z_{J-1}$

To solve minimization in step 2 and step 3 for the PAPR structural constraint, the “matrix nearness problems” [30] is used, whose algorithm is detailed in [24] (p. 198).

To summarize the framework in which alternating projection algorithm operates is shown in the ensuing diagram, Figure A2.
